# Integrating 3D Printing with Injection Molding for Improved Manufacturing Efficiency

**DOI:** 10.3390/polym17141935

**Published:** 2025-07-14

**Authors:** Zdenek Chval, Karel Raz, João Pedro Amaro Bennett da Silva

**Affiliations:** 1 Faculty of Mechanical Engineering, Regional Technological Institute, University of West Bohemia, Univerzitni 2732/8, 301 00 Plzen, Czech Republic; kraz@fst.zcu.cz; 2 Instituto de Matemática e Tecnologia St. Universitario, Federal University of Catalã, Campus I, Catalão 75704-020, GO, Brazil; joao_bennett@discente.ufcat.edu.br

**Keywords:** MJF, nylon, foam material, 3D printing

## Abstract

This study investigates a hybrid manufacturing approach that combines 3D printing and injection molding to extend the limitations of each individual technique. Injection molding is often limited by high initial tooling costs, long lead times, and restricted geometric flexibility, whereas 3D-printed molds tend to suffer from material degradation, extended cooling times, and lower surface quality. By integrating 3D-printed molds into the injection-molding process, this hybrid method enables the production of complex geometries with improved cost-efficiency. The approach is demonstrated using a range of polymeric materials, including ABS, nylon, and polyurethane foam—each selected to enhance the mechanical and thermal performance of the final products. Finite element method (FEM) analysis was conducted to assess thermal distribution, deformation, and stress during manufacturing. Results indicated that both temperature and stress remained within safe operational limits for 3D-printed materials. An economic analysis revealed substantial cost savings compared to fully 3D-printed components, establishing hybrid manufacturing as a viable and scalable alternative. This method offers broad industrial applicability, delivering enhanced mechanical properties, design flexibility, and reduced production costs.

## 1. Introduction

Growing pressure for rapid, economical, and highly customized production has driven parallel advances in both additive manufacturing (AM) and conventional fabrication. Although plastic injection molding continues to dominate mass production thanks to its repeatability and surface quality, its reliance on costly tooling, lengthy mold-development cycles, and limited geometric freedom remains a significant barrier for complex parts [1,2,3].

By contrast, AM, popularly known as 3D printing, delivers exceptional design latitude, enabling intricate shapes without hard tooling and excelling in rapid-prototype or low-volume runs [4,5]. Nevertheless, AM components often exhibit lower mechanical strength, reduced thermal tolerance, and less refined surface finishes, while the restricted material palette and long cooling times still hinder their use in structural, high-performance applications [6,7,8,9].

Hybrid manufacturing has emerged as a promising strategy for closing these performance and economic gaps. By integrating 3D-printed elements—especially powder-bed fusion parts produced via Multi Jet Fusion (MJF)—with injection molding, engineers can obtain complex, high-integrity parts while trimming costs and lead times [10,11]. MJF has been shown to outperform some other powder-bed fusion methods in terms of dimensional accuracy and consistency, which supports its viability in hybrid workflows [12,13,14,15].

This combination offers sharply reduced mold-making expenses, faster product-development cycles, and the freedom to create advanced geometries. Furthermore, printed shells can be back-filled with injection-molded polymers such as ABS (acrylonitrile butadiene styrene), PA (polyamide), PP (polypropylene), or PU (polyurethane) foam, thereby boosting tensile strength, stiffness, thermal insulation, and even fire resistance when required [16,17,18,19,20].

From an economic viewpoint, hybrid production is compelling because 3D-printed molds paired with injected polymer cores minimize material waste and overall expense while preserving design agility—an advantage eagerly sought in automotive, aerospace, and fast-moving consumer-goods sectors [21,22,23,24].

The recent literature reinforces these benefits: Mehrpouya et al. [5] demonstrated improved mechanical performance through multi-material powder-bed fusion; Issabayeva and Shishkovsky [3] argued that hybridization can offset FDM mechanical weaknesses; Chan et al. [6] linked hybrid workflows to shorter supply chains; and Boros et al. [25] described the limitation of 3D-printed injection molds. Other works have focused on improving part durability and accuracy, such as that of Yelamanchi et al. [26] in automotive applications and Liu et al. [27] on enhancing PA12 composites via post-processing. These studies collectively support the hybrid approach used in this paper.

Accordingly, this study evaluates hybrid parts consisting of MJF-printed shells and injection-molded polymer infills, employing finite element method (FEM) simulations to analyze thermal and mechanical behavior and comparing costs with conventional production routes to confirm the scalability and efficiency of the proposed process [28,29].

Combining 3D printing and injection molding is also limited by the internal core deformation during molding. These issues affect the accuracy and quality of internal features, as mentioned in [30].

## 2. Materials and Methods

This chapter is focused on a description of both materials used, the base 3D-printed frame and the internal filling.

### 2.1. MJF AM Production

This study utilizes the MJF 3D printing process, a powder-bed fusion technique developed by HP (Hewlett-Packard, Palo Alto, CA, USA) that differs fundamentally from traditional laser-based sintering methods. The printer used is HP MJF 4200. Instead of using a laser to directly sinter the material, MJF relies on the selective application of chemical agents combined with infrared radiation to achieve precise thermal control and material fusion [18,19]. The material employed in this research is PA12GB—a polyamide 12 composite reinforced with 40% glass beads—which offers improved dimensional stability, stiffness, and thermal resistance compared to standard PA12 [31].

The MJF process begins by evenly distributing a thin layer of powder across the build platform. This layer is preheated to a temperature just below the material’s melting point to optimize energy absorption during fusion [32,33]. A printhead then selectively deposits two types of chemical agents: a fusing agent, which promotes localized heat absorption, and a detailing agent, which controls the spread of heat and material melting at the boundaries of the part [34,35].

Once the agents are applied, an infrared lamp uniformly irradiates the surface. The areas treated with the fusing agent experience a localized rise in temperature beyond the melting point, causing the powder particles to coalesce. The detailing agent, in contrast, either inhibits or precisely controls fusion at the edges of the part to ensure clean, sharply defined boundaries [36,37,38]. After fusion, the build platform lowers by one layer of thickness, and the process is repeated iteratively until the complete 3D object is formed [39].

**Figure 1:**
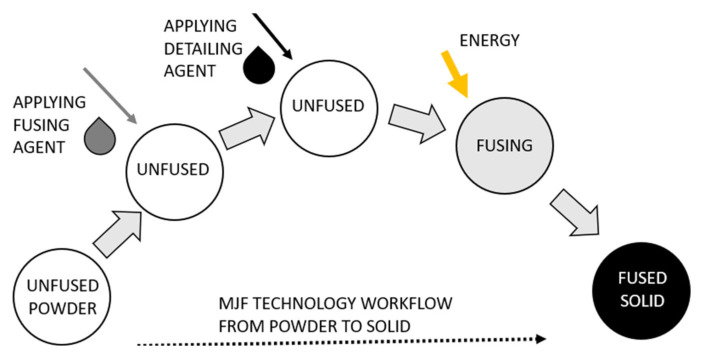
Principle of the MJF technology.

Figure 1 visually illustrates the MJF workflow. Initially, the powder material is spread across the work area. The fusing agent is then selectively applied to regions where particle bonding is required. Next, the detailing agent is deposited in areas where precise feature control or reduced fusion is desired—particularly at the part’s edges. Exposure to infrared radiation initiates fusion exclusively in the targeted zones, solidifying the structure layer by layer.

This innovative method enables the production of components with fine surface resolution, consistent mechanical properties, and complex geometries, making it particularly well-suited for hybrid manufacturing applications where both precision and performance are critical.

### 2.2. Polymeric Injection Materials Usable for Creating Hybrid Material

The following summary describes various materials that can be used as internal injection-molding materials. Their use will significantly advance the resulting properties of a product that was originally manufactured as a 3D-printed package. This allows for modification of its properties, as 3D printing itself has a significantly smaller range of available materials than injection molding [40].

- Polymethyl methacrylate (PMMA)

It is a thermoplastic material with good mechanical resistance, easy machinability, and easy processing. Common uses of this polymer are in decorative souvenirs, solar panels, and window protection [41].

- Acrylonitrile butadiene styrene (ABS)

It is one of the most popular injection-molding materials, has good mechanical resistance and chemical resistance to acid–base materials and oils, and has a lower price than other types of materials. It is commonly used in automotives and sports [42].

- Nylon (PA)

Polyamides generally have good mechanical properties. PA11 is a bio-based polymer and is resistant to UV radiation. PA12 polymers have greater abrasion resistance than PA11. PA46 is used in areas where its good mechanical resistance at high temperatures and chemical and electrical resistance is appropriate [43]. This material is commonly used for guitar strings, bearings, and gears.

- Polycarbonate (PC)

This material has good optical clarity, better than glass. This material is commonly used in protective goggles, face shields, and motorcycle helmet visors [44,45].

- Polyoxymethylene (POM)

POM is commonly used in parts with high precision requirements. It has high dimensional stability and rigidity. It is commonly used in automotive applications, gears, and bearings.

- Polypropylene (PP)

This material has heat and chemical resistance, is tough, and can be recycled many times, all of which make this material easy to use. This material is used in many applications today. However, this material has a high coefficient of thermal expansion, which makes it unsuitable for high-temperature applications [46,47].

- Polyethylene (PE)

This material has 3 subclasses: high-density polyethylene (HDPE), low-density polyethylene (LDPE), and polyethylene terephthalate (PET) [48]. The classes have been divided according to the respective density of each material. All types have good impact resistance and can withstand mechanical stress for a long time without deformation. The HDPE variant is usually used to make pipes and technical components. LDPE is more flexible; it is a good material for making shopping bags and packaging materials. PET is good for making bottles and other containers [49].

- Thermoplastic elastomer (TPE)

TPE is the only material used for injection molding that is made by mixing plastic and rubber. It cannot be used at high temperatures because it loses its original properties. In addition, it cannot be stretched for a long deformation.

### 2.3. Analysis and Possibilities of Using PU Foam as a Filler for Hybrid 3D-Printed Parts

Another option for creating hybrid materials is to use generally available polyurethane foam. This material is relatively inexpensive, and no special machinery is required for its application [50].

Polyurethane foam is a generally known adhesive and sealant material. It is widely used in the construction industry to fasten components such as doors and frames, for insulation, and for repairing various building structures. In the production of PU, a chemical reaction is carried out by mixing two monomers. The result of the chemical reaction of these products is the polymer itself [51].

The following tables show the basic general parameters of this material from the thermal aspect (Table 1) and the mechanical aspect (Table 2).

The mechanical properties of this material can be defined as follows:

**Table 2:** Mechanical properties of PU foam.

Compressive strength parallel to the direction of foam growth (MPa)	0.2
Compressive strength perpendicular to the direction of foam growth (MPa)	0.12
Tensile strength parallel to the direction of foam growth (MPa)	0.35
Tensile strength perpendicular to the direction of foam growth (MPa)	0.25
Shear strength (MPa)	0.16

The above tables show the general, mechanical, and thermal properties of PU expansion foam, and Table 3 provides a brief comparison of the mechanical properties of selected specific types of PU foams according to the datasheet provided by the manufacturer.

**Figure 2:**
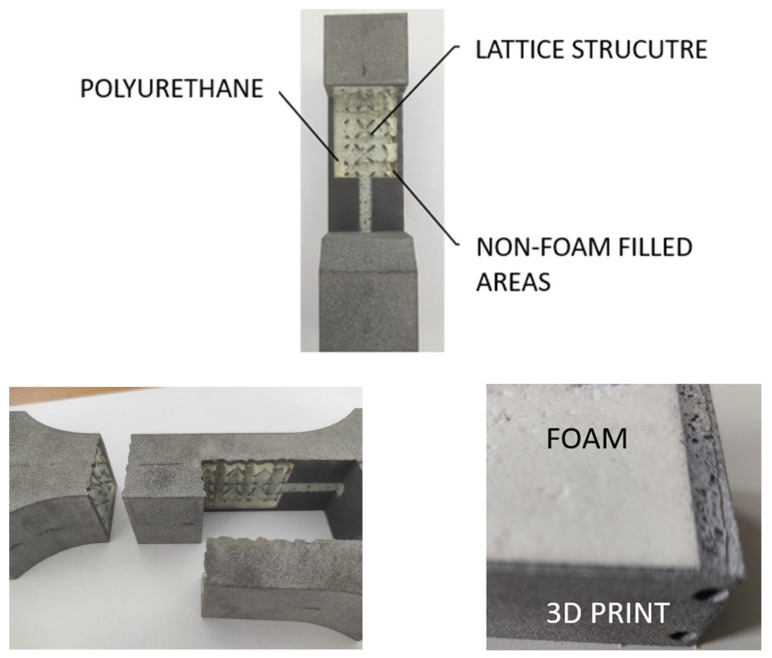
Examples of hybrid material—section view and detail.

The use of PU foam combined with 3D-printed molds (see Figure 2) can be a great alternative for creating hybrid parts, since the cost of creating 3D-printed molds is relatively low, and the price of PU foam is also not high. Therefore, using the combination of these two materials presents many advantages, such as the resulting mechanical properties of the printed materials combined with the various properties of the foams, such as insulation, fire protection, and others.

### 2.4. Reflection on Material Properties and Manufacturers

The selection of materials and their respective manufacturers plays a crucial role in the success of hybrid manufacturing strategies, as it directly influences mechanical performance, thermal resistance, and processing reliability. In this study, the primary material used for additive manufacturing is PA12GB (polyamide 12 with 40% glass beads), manufactured by Hewlett-Packard (Palo Alto, CA, USA) for use in their Multi Jet Fusion (MJF) systems. This material was chosen due to its proven thermal stability, dimensional accuracy, and enhanced rigidity, as supported by prior evaluations of its mechanical durability and process consistency [52].

The injection materials span a range of thermoplastics, including ABS, nylon (PA11, PA12), PP, and PU foam, sourced from commercial suppliers such as BASF (Ludwigshafen, Germany), SABIC (Riyadh, Saudi Arabia), and Sika (Baar, Switzerland) (e.g., Sika Boom). These materials were selected based on their availability, application-specific properties, and compatibility with hybrid processes. For instance, polypropylene offers excellent recyclability and toughness at a competitive cost, while PU foams (such as HandiFoam^®^ E84 (ICP Building Solutions Group, Norton OH, USA) and PU Foam Light (Arkema, Middleton, WI, USA)) provide a lightweight solution with strong insulating and energy-absorbing capabilities [25].

Crucially, these materials were not only chosen based on datasheet specifications, but also based on their industrial reputation and previous validation in mechanical and thermal simulations. The FEM analyses demonstrated that stress levels in hybrid components remained within safe bounds—even under elevated temperatures—validating both the material choice and the simulation fidelity.

The use of branded materials from trusted manufacturers ensures repeatability and traceability in production, which is essential for scaling hybrid manufacturing into industrial contexts. As materials such as PA12GB continue to evolve—often with tailored fillers or surface treatments—the scope for achieving even finer mechanical control in hybrid parts will expand, opening new avenues in aerospace, automotive, and functional prototyping sectors.

### 2.5. Process Modeling Methodology

To ensure accurate predictions of mechanical and thermal responses during hybrid part production, the process modeling relied on the finite element method (FEM) using a combined simulation workflow. The thermal and structural behavior of the printed and injected materials was modeled using a coupled transient analysis in Autodesk Moldflow 2025 (Autodesk, Inc., San Francisco, CA, USA) and Digimat-AM 2022.1 (Hexagon, Surrey, UK). The governing equations include Fourier’s law for heat conduction and the generalized Hooke’s law for elastic deformation under thermal and mechanical loads. For thermal analysis, the heat conduction equation was used as follows (1).

(1) $$q\dot{x}=-k\;d_{y}\;d_{z}\;(\partial T/\partial x)$$

The *dx*, *dy,* and *dz* are dimensions of the infinitesimal volume element in the respective direction. The heat transferd through the element in the x-direction is $q\dot{x}$. The generalized Hooke’s law is as follows (2).

*σ_ij_* = *C_ijkl_*
*ɛ_kl_* (2)

where *σ_kl_* represents the stresses and *ε_kl_* the strains, and *C_ijkl_* is a fourth-order tensor of the elastic modulus. This modeling approach enabled a detailed analysis of internal stresses, deformation modes, and thermal gradients, which were validated against material datasheets and typical process outcomes. Future improvements will involve coupling the injection simulation with in situ monitoring data to improve prediction fidelity.

## 3. FEM Analysis and Validation of Production

Hybrid manufacturing can be broadly described as the process of injecting polymer material into a 3D-printed mold, which simultaneously functions as the final product. The following section of this study examines the critical technical parameters involved in this process, with a focus on thermal profiles, material deformation, and stress distribution during production. These factors are analyzed using advanced mold flow simulations based on the finite element method (FEM) to ensure accurate prediction of material behavior and structural integrity. The process parameters for the production, such as temperature or pressure, were selected according to the datasheet of each material as a middle-range value. The suitable simulation tool was a combination of the AM simulation tool, such as Digimat-AM, and a mold flow simulation, such as Autodesk MoldFlow. These tools were used for understanding the maximal Von Mises stress with respect to the strength of the material under the actual temperature. The displacement shows the maximal change of position of individual nodes of the model during the production process.

**Figure 3:**
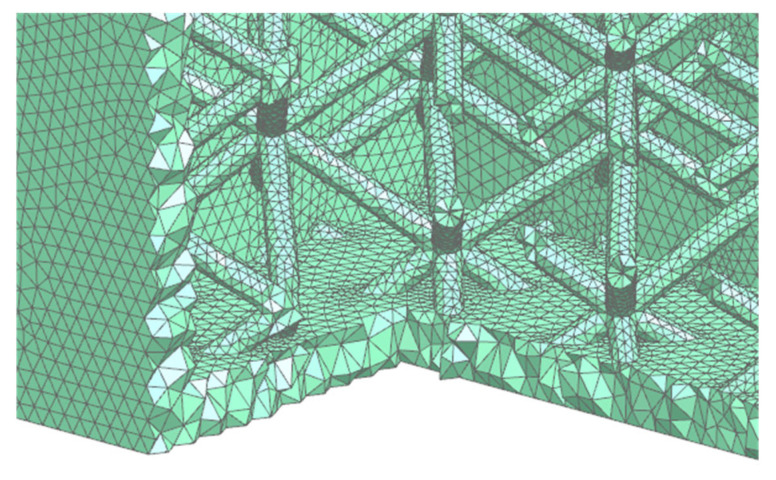
Detail of the FEM model.

The important aspect of the model is the FEM model. The model used mainly tetrahedral 10-node elements with a boundary layer. The number of elements was 1,850,357. Figure 3 shows the detail (section view) of the mesh.

### 3.1. Thermal Load During Injection Molding

**Figure 4:**
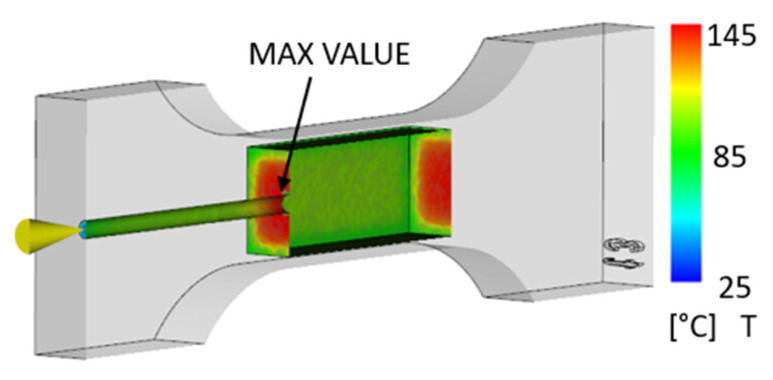
Display of the temperature field at the interface of the injected material and the 3D-printed envelope [°C].

The simulation considered filling the internal cavity of a 3D-printed part with polypropylene as an example. The injection temperature was 220 °C, and the injection pressure was 72 MPa. The plastic 3D-printed part (mold) was 22 °C. From Figure 4, it is clear that the temperature inside the cavity before the thermal degradation of the 3D-printed material did not exceed 150 °C.

**Figure 5:**
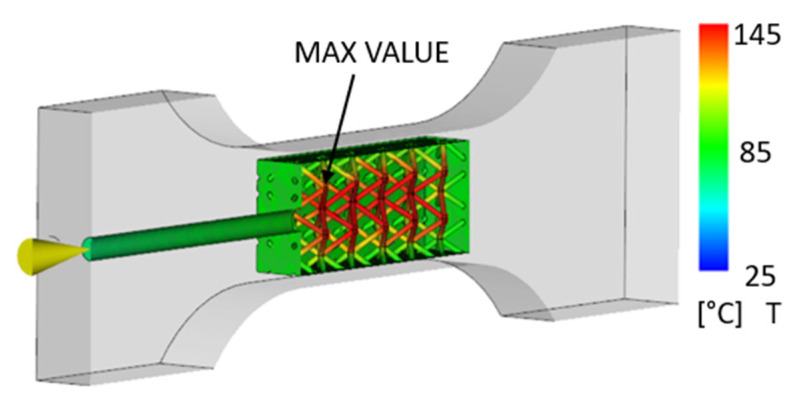
Display of the temperature field at the interface of the injected material and the 3D-printed envelope with an internal lattice structure [°C].

The simulation was performed on two different parts (with and without an internal lattice structure) so that they could be compared. Figure 5 shows the thermal distribution for the part with a lattice structure in its internal cavity to improve strength and structural properties.

It can be noted that the results for the part with and without the internal lattice structure were comparable. The maximum temperature inside the part during polypropylene injection molding was approximately 145 °C. This value is correct, since the melting point of the 3D-printed material PA12GB is 186 °C according to the datasheet provided by the manufacturing company [47,48].

### 3.2. Deformation Caused by Injection Molding

**Figure 6:**
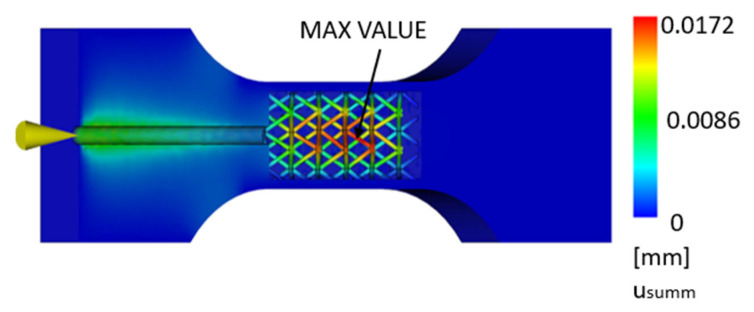
Maximum displacement for a part composed of a 3D-printed envelope with an internal lattice structure and an injection-molded core [mm].

**Figure 7:**
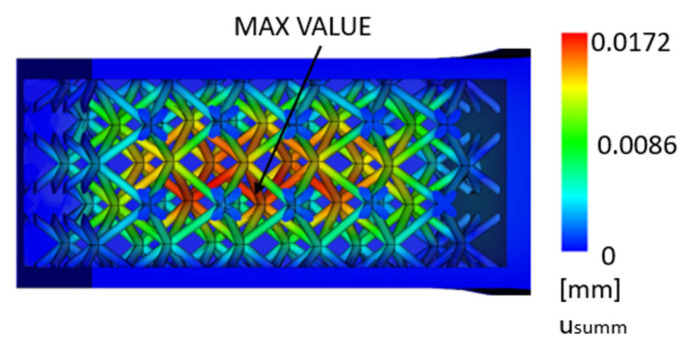
Maximum displacement for a part composed of a 3D-printed envelope with an internal lattice structure and an injection-molded core—detail [mm].

In the simulation, it can be observed that the 3D-printed part is subject to relatively small displacement in the area where the polymer is injected, especially in the area of the inner cavity wall. The deformation is more significant in the inner lattice structure, which is shown in Figure 6 and Figure 7.

This deformation is caused by the fact that the presence of an internal lattice structure makes it difficult to fill the molded material, and, due to filling, this internal lattice structure deforms under the pressure of the plastic.

### 3.3. Strength Analysis of Hybrid Material Production Technology

**Figure 8:**
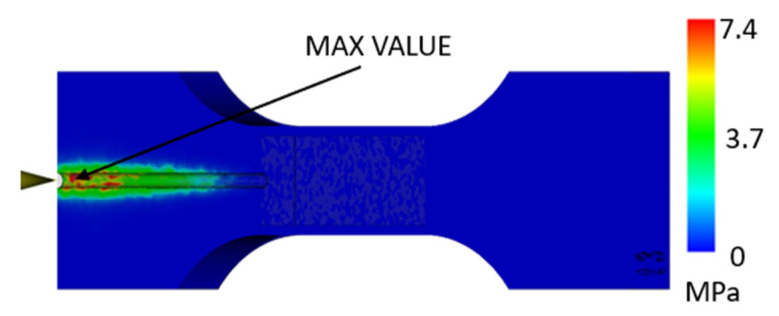
Maximum Von Mises stress for a part composed of injection-molded material and a 3D-printed envelope [MPa].

In addition to the displacements, a stress analysis was performed on the printed part, and, again, two simulations were performed, one for the part without a lattice structure and the other with a lattice structure. For the part without a lattice (Figure 8), it was possible to observe that more significant stress occurs only in the gate area due to the pressure exerted during injection molding.

**Figure 9:**
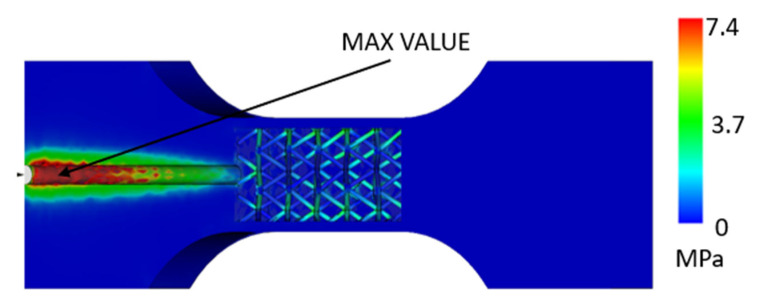
Maximum Von Mises stress in a part composed of injection-molded material and a 3D-printed envelope with a lattice structure [MPa].

**Figure 10:**
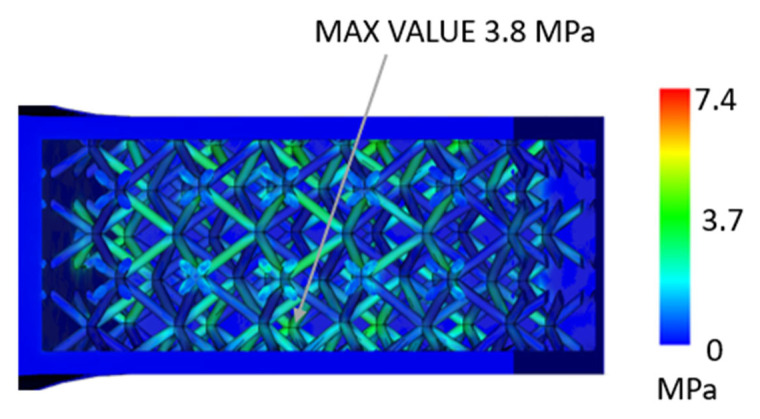
Maximum Von Mises stress in a part composed of injection-molded material and a 3D-printed envelope with a lattice structure—detail [MPa].

In the case of a part with a lattice structure (Figure 9 and Figure 10), it can be seen that in the cavity near the injection area, the pressure reaches a maximum value of around 7 MPa, and in the internal structure, it can be seen that there is a stress of around 4 MPa. According to the datasheet, the maximum stress that the material can withstand is 30 MPa, which means that the material is able to survive the injection-molding process, even assuming its heating and the associated changes in mechanical properties.

### 3.4. Economic Evaluation of the Use of Hybrid Materials

**Figure 11:**
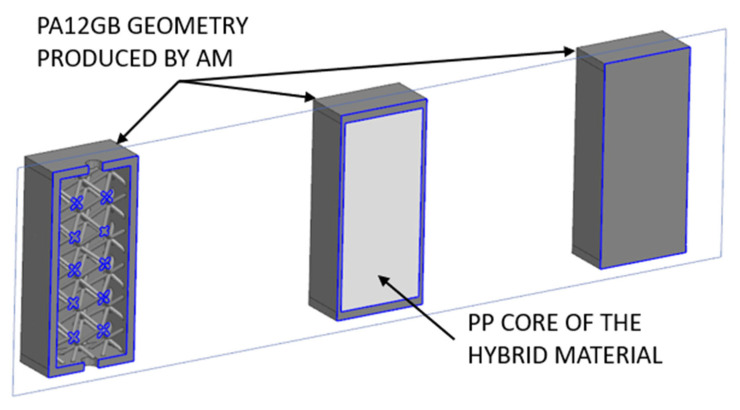
Different central areas of specimens from previous research, used for economic evaluation.

An important aspect of the usability of different types of 3D-printed and injection-molded parts is the economic evaluation. For comparison, theoretical parts are used (Figure 11). The parts are in cross-section, the 3D-printed material in solid state is shown in dark gray, and the core of the hybrid material is in the form of injected plastic applied to the sample with a lattice structure.

The economic model compares the material and production costs of various part configurations—including fully 3D-printed, lattice-filled, and hybrid-injected parts—based on material prices, part volume, and estimated production time. A completely full 3D-printed sample has a production cost of USD 30.17.

A 3D-printed sample with an internal lattice structure has a production cost of USD 20.32. The price of a hybrid part with a lattice structure and PP injection is USD 20.45.

The price analysis of the hybrid part was based on the assumption that the price of the hollow part into which the injection-molded material will be applied is approximately USD 20.32. In the chosen benchmark example, the weight of the applied injection-molded material is 42.72 g [49,50]. The price analysis considers the use of an estimated PP price of 3.28 USD/kg (the value is very dependent on the chosen material). The value of the material to be injected would therefore be approximately USD 0.13, regardless of the energy and work in the injection process [51,52]. The summary is shown in Table 4. There is a significant time dependency for these technologies; for example, the production time for an AM batch can vary from 6–8 h, and the injection molding is shorter than 1 min per part.

## 4. Discussion

The results confirm that hybrid manufacturing using MJF-printed molds and injection-molded polymers offers strong technical and economic advantages. FEM simulations showed that thermal loads during injection remained well below the degradation threshold of PA12GB, confirming the process’s thermal safety and material compatibility. Displacement and stress analyses indicated minimal deformation and low internal stress concentrations, even under high injection pressures, suggesting strong mechanical stability. Thermal and stress simulations are consistent with findings by Liu et al. [20], who also observed a high thermal stability of PA12-based components.

Cost analysis revealed a 30–40% savings compared to full 3D printing, supporting conclusions by Chan et al. [6] and Boros et al. [25] regarding hybrid manufacturing’s efficiency and reduced lead times. The mechanical performance remained stable even when using low-cost fillers like PU foam, which is in line with the multifunctional applications discussed by Farrugia [9].

Compared to studies like those of Boros et al. [25] and Podsiadły et al. [33], our approach further validates that hybridization improves strength and material diversity, overcoming common AM limitations. These results highlight the potential for scalable, functional hybrid components in industrial applications.

## 5. Conclusions

This research demonstrates the potential of combining 3D printing and injection-molding technologies to create hybrid materials that capitalize on the advantages of both methods while mitigating their respective limitations. Through the integration of the MJF 3D printing with various injection-molding materials, a cost-effective, flexible, and efficient manufacturing process can be achieved. Unlike injection molding alone, which requires expensive tooling and limits design complexity, the hybrid method supports rapid prototyping and small-batch manufacturing without compromising mechanical strength.

The findings reveal that hybrid materials provide enhanced mechanical, thermal, and economic properties compared to standalone 3D-printed or injection-molded parts. The use of polymeric fillers, such as polypropylene and polyurethane foam, contributes to improved structural integrity, insulation, and fire resistance. Additionally, the finite element method (FEM) simulations confirmed the feasibility of hybrid production, with stress and deformation levels well within acceptable limits for the materials tested.

Economic analysis further supports the viability of hybrid parts, showing significant cost savings compared to fully 3D-printed components while maintaining comparable performance. For instance, hybrid parts with lattice structures and injected cores are more affordable and resource-efficient than solid 3D-printed parts, highlighting their practicality for industrial applications.

In conclusion, hybrid manufacturing holds significant promise for industries such as automotive, aerospace, and construction, where tailored material properties, cost efficiency, and design flexibility are critical. Future research should explore additional material combinations, optimization of internal lattice designs, and the long-term durability of hybrid parts to further expand their application potential. The research is going to continue with mechanical testing and exploring all the mechanical properties of these materials.

## Figures and Tables

**Table 1:** Thermal properties of PU foam.

Thermal conductivity	W/mK	0.018–0.023
Density	kg/m^3^	>30
Max long-term temp.	°C	110–130

**Table 3:** Properties of selected PU foams.

Material	Density (kg/m^3^)	Compressive Strength (MPa)	Tensile Strength (MPa)
HandiFoam^®^ E84 Class 1(A) II-16 Spray Foam	28–34	Parallel 0.182; perpendicular 0.11	0.172
Sika Boom	20	0.05 (with 10% of deformation)	0.1
PU Foam HH	25–30	0.02 (with 10% of deformation)	0.11
Rigid Foam PU	30	Parallel 0.14–0.18; perpendicular 0.13–0.18	0.35
PU Foam Light	62	Parallel 0.64; perpendicular 0.41	Parallel 0.79 Perpendicular 0.44

**Table 4:** Costs summary.

Type of specimen	Costs (USD)
Full 3D-printed sample	30.17
A 3D-printed sample with an internal lattice structure	20.32
A hybrid part with a lattice structure and PP	20.45

## Data Availability

The original contributions presented in this study are included in the article. Further inquiries can be directed to the corresponding author.

## Abbreviations

The following abbreviations are used in this manuscript:

MJF	Multi Jet Fusion
USD	Dollar
PU	Polyurethane
HP	Hewlett Packard
AM	Additive Manufacturing
ABS	Acrylonitrile Butadiene Styrene
PA	Polyamide
PP	Polypropylene
